# INTEREST GROUP SESSION—INTERGENERATIONAL LEARNING, RESEARCH, AND COMMUNITY ENGAGEMENT: USING INNOVATIVE INTERGENERATIONAL STRATEGIES TO BOOST CAREERS IN AGING

**DOI:** 10.1093/geroni/igz038.2098

**Published:** 2019-11-08

**Authors:** Joann M Montepare, Laura K Donorfio

**Affiliations:** 1 Lasell College, Newton, Massachusetts, United States; 2 University of Connecticut, Waterbury & Storrs, United States

## Abstract

Populations are aging dramatically, and call for higher education to be more age-friendly and pave the way for career paths in aging. The Careers in Aging Week (CIAW) program sponsored annually by the Academy for Gerontology in Higher Education (AGHE) of GSA has been a core stimulus for building career interest – however, more could be done to strengthen and invigorate this effort. This symposium will show how intergenerational exchange can be used to mount interest in careers in aging and create new pipelines to gerontology programs in higher education. Examples of innovative approaches will demonstrate how career information can be communicated to students in more creative and compelling ways. The first paper will set the stage with an evidence-based overview of emerging areas for career development, and a presentation of career planning models to aid student understanding as to how to make aging career decisions. Two presentations will then focus on different aging-workforce initiatives aimed at building educational pipelines that connect high-school students with college students studying gerontology. In addition to highlighting the oft-overlooked population of high school students, attention will be given to the importance of including minority student populations in career development efforts. The final paper will describe the utility of broader intergenerational strategies that build bridges across students, educators, aging professionals, and community collaborators via campus career events. The discussant will bring these efforts together with an intergenerational programming lens that higher education can use to amplify awareness about the wide-range of career opportunities aging offers.

